# From digital stimulus to hiking participation: an SOR-based examination of the internal associations between social media hiking content and participation intention in Chinese generation Z consumers

**DOI:** 10.3389/fpsyg.2026.1877548

**Published:** 2026-07-20

**Authors:** Tian Yin, Yanfeng Peng, Zetian Wang, Xu Liu, Jiale Wang, Tiande Pan

**Affiliations:** 1 School of Physical Education, Yunnan Minzu University, Kunming, Yunnan, China; 2 Xinjiang Normal University, Urumqi, Xinjiang, China; 3 Hezhou University, Hezhou, Guangxi, China

**Keywords:** destination image, generation Z consumers, network analysis, participation intention, social connectedness, social media hiking content, travel inspiration

## Abstract

**Objective:**

This study examined the relationship between perceived social media hiking content and hiking participation intention among Chinese Generation Z consumers. It further tested whether this relationship was consistent with a serial mediation pattern involving travel inspiration and destination image, and whether social connectedness moderated these associations. Node-level network analysis was also used to identify key relational structures among construct items and dimensions.

**Methods:**

An online questionnaire survey was conducted among 1,186 Chinese Generation Z consumers with prior contact with social media hiking content. Participants completed measures of perceived social media hiking content, travel inspiration, destination image, social connectedness, and hiking participation intention. SPSS 26.0 was used for common method bias testing, descriptive statistics, and correlation analysis. The PROCESS macro was used to examine serial mediation and moderated conditional process models. R software was used to estimate an undirected node-level network and identify conditional associations among items and dimensions.

**Results:**

Perceived social media hiking content was significantly and positively associated with hiking participation intention. The serial mediation analysis showed that this relationship was consistent with indirect pathways involving travel inspiration and destination image. Social connectedness moderated some of the associations. At higher levels of social connectedness, the positive associations among perceived social media hiking content, destination image, and hiking participation intention were stronger, and the indirect associations through destination image and through the travel inspiration–destination image pathway were more pronounced. Network analysis indicated that hiking participation intention and destination image were the main cross-community connection areas. Future participation likelihood, affective image, adoption intention, recommendation intention, and infrastructure evaluation emerged as key bridge nodes.

**Conclusion:**

Perceived social media hiking content was positively associated with hiking participation intention among Chinese Generation Z consumers, and this relationship was consistent with a serial mediation pattern involving travel inspiration and destination image. Social connectedness moderated some of these associations, suggesting that the link between perceived hiking-related social media content and hiking participation intention may vary according to consumers’ relational embeddedness. Overall, this study provides correlational evidence for applying the stimulus–organism–response framework to leisure sport consumption and digital tourism behavior, and offers practical insights for social media hiking content communication and outdoor leisure consumption.

## 1 Introduction

Social media has become deeply embedded in the information ecology of contemporary consumers. It now serves as a major digital medium through which individuals obtain information, express interests, and engage in emotional interaction (Kapoor et al., 2018). According to DataReportal, global social media user identities reached 5.66 billion in October 2025, equivalent to 68.7% of the world’s population (Kemp, 2025). In China, the number of internet users reached 1.123 billion by June 2025, with an internet penetration rate of 79.7% (CNNIC, 2025). With the expansion of short-video platforms and algorithmic content recommendation, social media has moved beyond interpersonal communication. It has become a key arena in which everyday lifestyles are displayed, observed, and imitated (van Dijck and Poell, 2013). Prior research has also shown that social media is closely related to how consumers understand, evaluate, and participate in consumption activities (Le and Ngoc, 2024). Thus, social media is not only a channel for information dissemination but also a digital environment linked to consumption imagination, lifestyle identification, and behavioral intention.

This transformation is especially visible in outdoor leisure contexts. Hiking routes, natural landscapes, personal experiences, and practical tips are highly visible in social media content, where hiking is often presented as accessible to ordinary consumers. Hiking is no longer presented only as a niche outdoor practice requiring specialized skills; it is increasingly represented as a leisure sport activity that can be watched, imagined, and potentially tried (Han et al., 2022). In this study, platform-based content centered on natural scenery, embodied outdoor experience, route information, and personal narratives is defined as social media hiking content (SMHC). In the social media context, audience responses to SMHC are not limited to platform-level exposure. They also involve subjective evaluations formed through previous encounters with such content, including perceived attractiveness, authenticity, usefulness, credibility, and action guidance. Accordingly, this study examines SMHC from the perspective of audience-perceived evaluation. Unless otherwise stated, SMHC in this article refers to perceived social media hiking content rather than objective exposure frequency or platform-recorded viewing behavior.

Compared with traditional destination promotion, SMHC provides not only route, scenery, and experience information but also vivid visual scenes and peer narratives. These features may be associated with a lower psychological distance from outdoor activities (Nguyen and Tong, 2023) and with potential consumers’ interest in hiking destinations, destination-related imagination, and participation intention (PI) (Fang et al., 2023). At the same time, SMHC is often embedded in social interaction contexts, such as likes, comments, reposts, saves, and peer experience sharing. Its meaning therefore does not arise from content features alone, but also from the viewer’s social relationships, group connections, and relational embeddedness. This social dimension is particularly relevant to Generation Z consumers, who were born between 1997 and 2012 and have grown up in a highly platformized digital environment (Dimock, 2019). For this group, social media is an important source of consumption information, lifestyle identification, and leisure preference expression.

Beyond this social media context, the specific nature of hiking also makes it theoretically important. Hiking is neither a fully professionalized extreme sport nor a form of conventional sightseeing tourism. Rather, it lies at the intersection of physical activity, nature-based experience, and leisure consumption (Bratman et al., 2019). Walking in natural environments has been linked to stress recovery, emotional regulation, and psychological well-being (Pretty et al., 2005). From a consumption perspective, hiking-related activities are also expanding from individual leisure choices into composite consumption scenes involving destinations, routes, equipment, platform content, and experience services. This shift reflects growing consumer demand for experiential, health-oriented, and nature-based leisure activities (Pomfret, 2006). Taken together, these features make hiking a useful context for examining how perceived social media content is associated with intention toward an offline outdoor activity that involves both destination evaluation and bodily participation.

Although previous studies have linked social media content to tourism and outdoor recreation intentions, they have mainly examined user-generated content, information quality, trust, travel planning, and destination choice (Ayeh et al., 2013; Zeng and Gerritsen, 2014; Wang and Yan, 2022). Other studies have identified inspiration-related pathways between tourism short videos and travel intention or examined social connectedness in relation to outdoor recreation intention (Fang et al., 2023; Wu and Ding, 2023; Hung and Liou, 2023). These studies establish the broader relationships among digital content, psychological evaluation, and behavioral intention. However, they provide less clarity on how these relationships are configured when the response concerns participation in hiking rather than general destination visitation. Hiking participation intention involves anticipated engagement in a physically enacted outdoor activity, whereas perceived SMHC includes evaluations of content authenticity, usefulness, credibility, and action guidance. The hiking context therefore shifts the focus from destination-oriented information and visit intention to participation-relevant content appraisal and intended adoption of an outdoor leisure practice.

More specifically, it remains unclear whether travel inspiration (TI) and destination image (DI) make functionally distinguishable contributions to the association between perceived SMHC and hiking participation intention, and whether these associations vary across levels of social connectedness (SC). The theoretical gap therefore concerns the internal differentiation and social boundary conditions of the content-intention association, rather than the existence of this general association itself. Accordingly, this study examines the relationships among SMHC, TI, DI, SC, and PI among Chinese Generation Z consumers.

To organize these relationships, this study draws on the stimulus–organism–response (SOR) framework (Eroglu et al., 2001). The SOR framework proposes that external environmental cues are related to individuals’ cognitive, affective, or motivational states, which in turn are associated with attitudinal, intentional, or behavioral responses (Jacoby, 2002). In the present study, SMHC is conceptualized as a stimulus-related content cue within the SOR framework, whereas TI and DI are conceptualized as organism-level psychological evaluation variables. TI is conceptualized as an action-oriented motivational response involving new ideas, emotional activation, and approach-oriented tendencies associated with external content cues (Thrash and Elliot, 2003; Böttger et al., 2017). DI is conceptualized as a broader cognitive–affective appraisal of destination attributes and affective value and has been closely associated with tourism choice and visit intention (Baloglu and McCleary, 1999). In addition, SC may serve as an important boundary condition because individuals’ sense of connection with others and groups can be related to how they receive social information, platform interaction, and lifestyle-oriented content.

In summary, this study contributes to the literature in three respects. First, it specifies the application of an established SOR framework when the response is intention to participate in hiking rather than general destination visitation. By conceptualizing SMHC as perceived content appraisal rather than objective exposure, the model connects content evaluation with intended participation in an offline outdoor activity. Second, it differentiates TI as an action-oriented motivational response from DI as a broader cognitive–affective destination appraisal, while treating their serial ordering as a theoretical proposition rather than a causal conclusion. Third, it examines SC as a boundary condition under which the associations of perceived content and destination appraisal with PI may vary. The contribution therefore lies in specifying the domain, internal differentiation, and social boundary conditions of an established SOR framework, rather than proposing a new stimulus–organism–response sequence.

## 2 Theoretical framework and hypotheses development

### 2.1 Stimulus-organism-response (SOR) model

The SOR model provides a classic theoretical perspective for understanding the relationships among external environmental cues, individuals’ internal psychological states, and response tendencies. The model was first proposed by Woodworth (1929) and was later extended by Mehrabian and Russell (1974) to environmental psychology and consumer behavior research. It emphasizes that the relationship between external environmental cues and individuals’ attitudes, intentions, or behavioral responses should be understood in relation to their cognitive, affective, or motivational states (Woodworth, 1929; Mehrabian and Russell, 1974). Prior studies have applied the SOR model to online consumption and social media contexts, suggesting that platform content, interaction cues, and informational stimuli are associated with purchase, participation, or continuance intentions through consumers’ internal psychological states (Chang et al., 2011; Ul Islam and Rahman, 2017). For example, Le and Ngoc (2024) treated social media peer communication as the stimulus, perceived brand quality and brand preference as organism-level responses, and online shopping intention as the final response. This application further indicates that the SOR framework is useful for organizing the relationships among stimulus-related cues, psychological evaluation variables, and intentional responses in social media contexts.

Following this theoretical logic, the present study conceptualizes SMHC as the external stimulus, TI and DI as sequential organism-level psychological states, and PI as the response variable. Specifically, SMHC presents experiences, practical knowledge, and landscapes through short videos, images, and personal narratives. Such content may be associated with new ideas, emotional arousal, and action tendencies, which may in turn be related to consumers’ overall evaluations of the natural environment, atmosphere, and affective value of hiking destinations, as well as their PI (Pike, 2002; Stylidis et al., 2017; Thrash and Elliot, 2004). In addition, this study incorporates SC into the SOR framework as a boundary condition. Individuals’ sense of belonging and relational embeddedness may be related to how they interpret social cues in social media content and whether they perceive SMHC as shareable, imitable, and participatory lifestyle information (Williams, 2006).

### 2.2 Direct association between SMHC and PI

In this study, SMHC refers to Generation Z consumers’ perceived evaluation of hiking-related social media content they had previously encountered, including its attractiveness, authenticity, usefulness, credibility, and action guidance. It is conceptualized as a perceived content-related stimulus rather than as objective exposure frequency or browsing duration. PI refers to consumers’ self-reported likelihood of participating in hiking activities, trying hiking routes, or recommending hiking destinations in the future, and is conceptualized as an intention-based response rather than actual hiking behavior. This conceptualization is consistent with the theory of planned behavior, which views behavioral intention as a proximal indicator of future behavior (Ajzen, 1991).

In social media environments, content is not an isolated piece of information but a socially embedded cue. Its credibility may depend on content features, source cues, update recency, and communication context (Westerman et al., 2014; Jenkins et al., 2020). Trust in social media communication is also related to interpersonal relationships, prior interaction, and information transparency (Cheng et al., 2017). Accordingly, when Generation Z consumers encounter SMHC, they may evaluate whether hiking is authentic, feasible, and worth considering through images, short videos, comments, saves, and sharing cues.

The association between SMHC and PI can be understood through uncertainty reduction and social learning. Uncertainty reduction theory suggests that individuals seek information to reduce ambiguity when facing unfamiliar or potentially risky situations (Berger and Calabrese, 1975). Hiking involves route choice, physical effort, equipment preparation, and destination-related risk perception. Therefore, route feedback, experience sharing, and peer evaluations on social media may provide concrete reference points for assessing whether hiking is manageable. Social media tourism information quality has been found to be positively associated with travel intention, with self-congruity and trust mediating this relationship (Wang and Yan, 2022). UGC has also been shown to be positively related to destination imagery and tourist visit intentions (Aboalganam et al., 2025). These findings suggest that perceived hiking-related social media content may be associated with PI through information quality, credibility, trust, and destination-related imagery.

SMHC may also be linked to PI through social learning processes. Social learning theory emphasizes that individuals can learn from observing others’ experiences and outcomes (Bandura, 1977). Through visualized and contextualized presentations of hiking, SMHC allows viewers to observe how others prepare for, experience, and evaluate hiking activities. Such content may make hiking appear more imitable, participatory, and lifestyle-relevant. Therefore, SMHC is expected to be positively associated with PI among Generation Z consumers. Given the cross-sectional design of this study, this hypothesis is interpreted as an association rather than as evidence of causal influence. On this basis, the following hypothesis is proposed:

*H1*: SMHC is positively associated with PI.

### 2.3 Indirect association pathways involving TI and DI

TI may represent the first psychological pathway linking SMHC and PI. Drawing on inspiration theory, TI refers to an evoked motivational state in which individuals perceive new possibilities, experience emotional arousal, and develop approach-oriented action tendencies after encountering external cues (Thrash and Elliot, 2003, 2004). In this study, TI is conceptualized as Generation Z consumers’ psychological readiness to imagine and consider hiking in relation to previously encountered SMHC, rather than as general interest or enjoyment. In digital tourism contexts, short videos, image-based content, and user experience narratives have been associated with pre-experience formation and with further exploration or action (Tussyadiah et al., 2018; Fang et al., 2023). More recent empirical evidence further suggests that tourism short-video content is associated with customer inspiration and travel intention, and that inspiration-related states may form an indirect pathway between short-video content and travel intention (Wu and Ding, 2023). These findings support the present study’s view that TI may operate as a psychological pathway linking SMHC and PI. Based on this reasoning, the following hypothesis is proposed:

*H2*: TI mediates the association between SMHC and PI.

DI may serve as another psychological pathway linking SMHC and PI. DI refers to individuals’ overall representation of a destination formed through information cues, prior knowledge, and affective evaluation. It involves not only judgments of functional destination attributes, but also perceptions of atmosphere, emotional value, and symbolic meaning (Echtner and Ritchie, 1993; Gartner, 1994; Tasci and Gartner, 2007). In this study, DI is conceptualized as Generation Z consumers’ cognitive and affective evaluation of hiking destinations, including their perceptions of the natural environment, infrastructure, atmosphere, and affective value of hiking-related places. In social media tourism contexts, images, videos, comments, and experiential cues may be associated with destination attractiveness, perceived credibility, and experience expectations. Travel-related UGC has been shown to be associated with destination selection intention, with destination image examined as one of the mediating mechanisms (Nguyen and Tong, 2023). UGC has also been found to be positively related to destination imagery and tourist visit intentions, with destination imagery further associated with tourist visit intentions (Aboalganam et al., 2025). These findings inform the present framework by suggesting that social media-based hiking content may be associated with PI partly through consumers’ evaluations of hiking destinations. Accordingly, the following hypothesis is proposed:

*H3*: DI mediates the association between SMHC and PI.

TI and DI may further form a serial mediation pathway between SMHC and PI. This logic is consistent with both the SOR framework and destination image formation theory. External content cues may first be associated with an evoked motivational state and then with cognitive and affective evaluations of a specific destination. DI is also understood as a psychological representation formed through information sources, prior knowledge, and affective responses (Gartner, 1994; Baloglu and McCleary, 1999). TI usually emerges before destination evaluation becomes stable because it makes individuals aware of a desirable possibility that has not yet been experienced (Thrash and Elliot, 2004; Böttger et al., 2017). This inspired state may then be related to how consumers evaluate the natural environment, atmosphere, and affective value of hiking destinations. In this sense, TI and DI are not merely parallel psychological outcomes. Rather, they may reflect a sequential process from being inspired by SMHC, to evaluating hiking destinations more favorably, and finally to forming PI. Based on this reasoning, the following hypothesis is proposed:

*H4*: TI and DI serially mediate the association between SMHC and PI.

### 2.4 The moderating role of SC

SC refers to individuals’ subjective sense of belonging, closeness, and connection within interpersonal or group relationships (Baumeister and Leary, 1995; Lee and Robbins, 1995). In this study, SC is conceptualized as the degree to which Generation Z consumers perceive themselves as socially connected, supported, and embedded in interpersonal or group relationships. Rather than treating SC as an independent predictor of PI, this study positions SC as a boundary condition because the central question is whether the associations linking SMHC, DI, and PI vary across different levels of relational embeddedness.

This positioning is consistent with the social nature of SMHC. SMHC is often encountered in interactive settings involving likes, comments, reposts, saves, and peer experience sharing. Its meaning therefore does not come only from visual scenes, route information, or peer narratives, but also from the relational context in which viewers interpret and evaluate such content. Prior studies have shown that social media use is closely related to social capital and maintained social relationships (Ellison et al., 2007), and that social connectedness can also be derived from online social networking environments (Grieve et al., 2013). Research on virtual communities further suggests that group norms and social identity are closely related to consumer participation in online communities (Dholakia et al., 2004). These findings suggest that consumers with higher SC may be more receptive to peer narratives, social interaction cues, and lifestyle-oriented content embedded in SMHC.

Accordingly, SC may condition the association between SMHC and PI. For Generation Z consumers with higher SC, SMHC may be more readily interpreted as a socially shareable leisure cue, such as a basis for peer discussion, shared travel experience, or group identity expression. In contrast, consumers with lower SC may remain more focused on information viewing or content browsing. SC may also condition the association between DI and PI. DI reflects individuals’ cognitive and affective evaluations of a hiking destination, but a favorable DI may not be equally related to PI for all consumers. Tourism research suggests that DI is related not only to destination attributes, but also to affective attachment, social interaction, and self-expression needs (Jang et al., 2009). For consumers with higher SC, a positive DI may be more closely associated with PI when the destination is also imagined as a site for traveling with peers, gaining social recognition, or reinforcing belongingness.

Recent outdoor leisure research provides more direct support for this boundary-condition logic. Hung and Liou (2023) found that personal social connections moderated the relationship between social media use and outdoor recreation participation intention, suggesting that social connections can condition how social media-related experiences are associated with outdoor leisure intention. Therefore, SC is modeled as a moderator because the theoretical focus is not whether SC is directly associated with PI, but whether the SMHC–PI and DI–PI associations vary across levels of SC.

Based on this reasoning, the following hypotheses are proposed:

*H5*: SC positively moderates the association between SMHC and PI, such that the positive association is stronger when SC is higher.

*H6*: SC positively moderates the association between DI and PI, such that the positive association is stronger when SC is higher.

**Figure 1:**
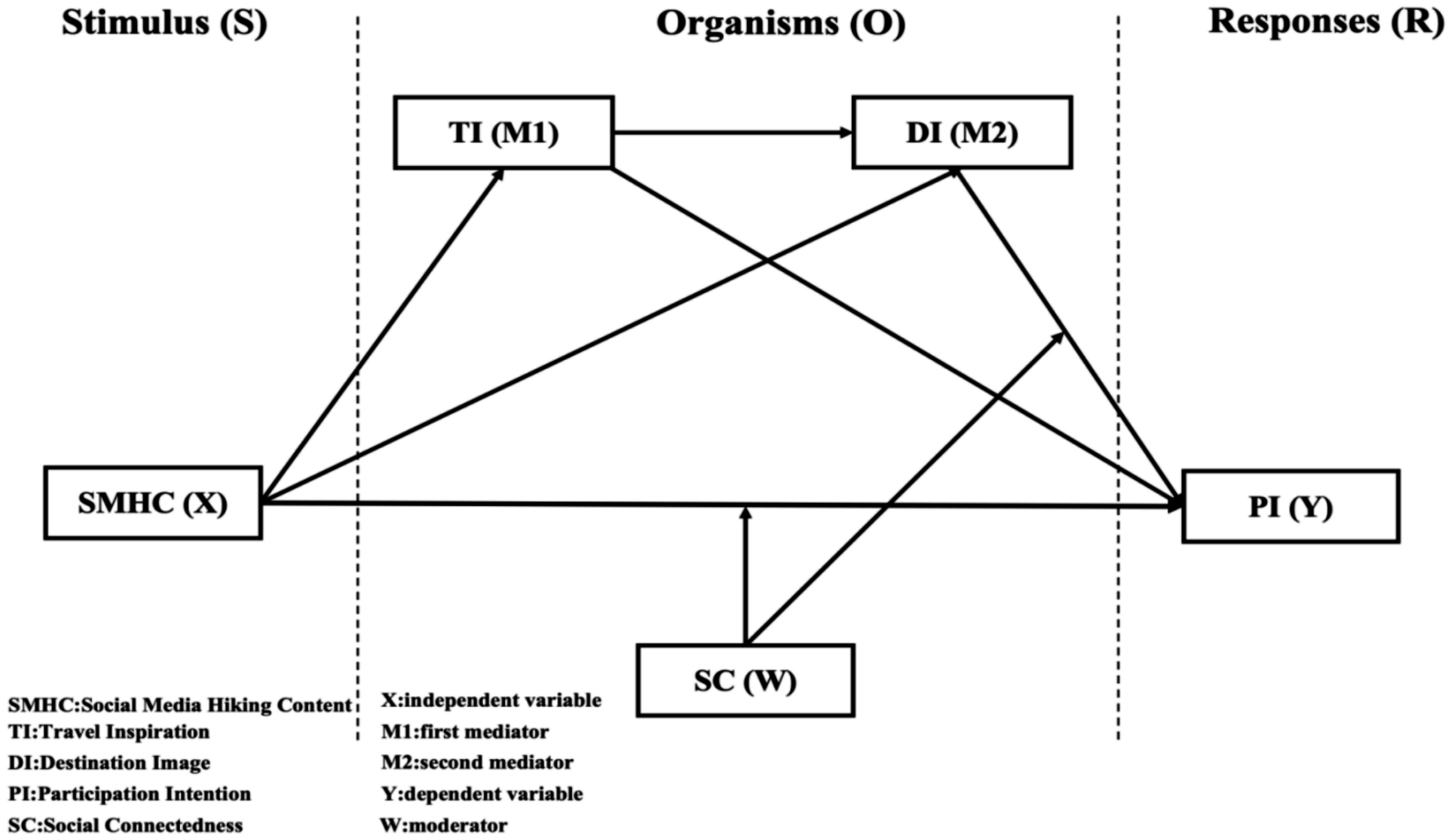
Hypothesized moderated serial mediation model.

Taken together, this study proposes a moderated mediation model (see Figure 1).

## 3 Methods

### 3.1 Participants

This study used convenience sampling to recruit Chinese Generation Z consumers who had previously encountered social media hiking content. Data were collected through an online questionnaire on Wenjuanxing beginning on January 10, 2026. Recruitment information was distributed through university student networks, social media platforms, and hiking-related online groups. The sample was mainly drawn from Yunnan, Sichuan, and Hubei Provinces. It should be noted that this recruitment approach yielded a non-probability sample defined by prior content exposure, which cannot represent the overall population of Chinese Generation Z consumers.

The study was approved by the Science and Technology Ethics Committee of Yunnan Minzu University before data collection began (Approval no. 2026015). All procedures followed the ethical principles of the Declaration of Helsinki. At the beginning of the online questionnaire, participants first read an introductory statement explaining the study purpose, data confidentiality, intended use of the data, voluntary nature of participation, anonymity, right to withdraw at any time, and minimal foreseeable risk. No monetary incentive or other compensation was provided. Only participants who provided informed consent were allowed to proceed to the questionnaire.

Participants were eligible if they met the following criteria: (1) aged 18–28 years; (2) had encountered social media hiking content within the past 3 months; (3) were able to read and complete the questionnaire independently; and (4) provided informed consent and participated voluntarily. Participants were excluded if they met any of the following criteria: (1) had taken part in high-intensity hiking within the past 2 years, such as a single-day outdoor hike exceeding 15 km, overnight camping, or similar activities; (2) were core members of an outdoor sports association or outdoor club, or were long-term intensive hiking participants; (3) showed obvious patterned responses, extremely short completion time, or substantial missing data; or (4) had a high missing rate for key variables or clear inconsistencies in questionnaire responses.

After data collection, the research team screened the questionnaires according to the predefined criteria. All researchers involved in questionnaire screening received unified training to ensure consistent data-quality assessment. After invalid responses were removed, 1,186 valid questionnaires were retained, yielding an effective response rate of 84.90%. The final sample included 823 males (69.4%) and 363 females (30.6%). Detailed demographic characteristics are presented in Table 1.

**Table 1:** Demographic characteristics of the sample (*N* = 1,186).

Variable	Group	*N*	%
Gender	Male	823	69.4
	Female	363	30.6
Age	18–20 years	546	46.0
	21–23 years	408	34.4
	24–28 years	232	19.6
Current status	Postgraduate student	214	18.0
	Undergraduate student	298	25.1
	Full-time employee	288	24.3
	Part-time / flexible worker	98	8.3
	Unemployed / job-seeking / others	288	24.3
Occupational field	Humanities, history, philosophy, and law	181	15.2
	Economics and management	95	8.0
	Science and engineering	283	23.9
	Medicine/agriculture	175	14.8
	Arts/sports	332	28.0
	Others	120	10.1
Monthly disposable expenses(RMB)	Below 1,000 CNY	58	4.9
	1,001–1,500 CNY	160	13.5
	1,501–2000 CNY	297	25.0
	2001–3,000 CNY	559	47.1
	Above 3,000 CNY	112	9.4

### 3.2 Measures

#### 3.2.1 Social media hiking content

The measurement of perceived social media hiking content (SMHC) was adapted from Du’s (2022) Adolescent Social Media Content Production Questionnaire and modified to fit the specific context of SMHC. It should be noted that SMHC in this study was not measured as objective platform-recorded exposure, browsing frequency, or time spent viewing content. Rather, it referred to participants’ subjective perceptions and evaluations of hiking-related social media content that they had encountered within the past 3 months. The scale assessed participants’ perceived SMHC, with items covering content attractiveness, authenticity, usefulness, credibility, and action guidance. The scale consisted of nine items rated on a 5-point Likert scale ranging from 1 = “strongly disagree” to 5 = “strongly agree.” Total scores ranged from 9 to 45, with higher scores indicating a higher level of perceived SMHC. In the present study, the scale showed good internal consistency, with Cronbach’s *α* = 0.927. The KMO value was 0.960. Confirmatory factor analysis indicated good construct validity: *χ*^2^/df = 2.374, CFI = 0.998, TLI = 0.991, RMSEA = 0.018, and SRMR = 0.011. In addition, SMHC demonstrated satisfactory composite reliability and convergent validity (CR = 0.928, AVE = 0.588), with a square root of AVE of 0.767.

#### 3.2.2 Participation intention

Participation intention (PI) was measured by adapting items from Chen and Tsai (2007) to the SMHC context. The scale assessed participants’ willingness to experience outdoor hiking routes, participate in hiking activities, choose hiking as a leisure or travel option, and recommend hiking destinations to others. It included five items rated on a 5-point Likert scale ranging from 1 = “strongly disagree” to 5 = “strongly agree.” Total scores ranged from 5 to 25, with higher scores indicating stronger PI. In this study, the scale demonstrated good internal consistency, with Cronbach’s *α* = 0.851. The KMO value was 0.866. Confirmatory factor analysis showed acceptable construct validity: *χ*^2^/df = 1.821, CFI = 0.970, TLI = 0.974, RMSEA = 0.044, and SRMR = 0.038. In addition, PI demonstrated satisfactory composite reliability and convergent validity (CR = 0.853, AVE = 0.539), with a square root of AVE of 0.734.

#### 3.2.3 Travel inspiration

Travel inspiration (TI) was measured by adapting the customer inspiration scale developed by Böttger et al. (2017) to the SMHC context. The scale included two states, “inspired-by” and “inspired-to,” and comprised 10 items. It was used to assess the new ideas, imagination, and action motivation reported in relation to previously viewed SMHC. Items were rated on a 5-point Likert scale ranging from 1 = “strongly disagree” to 5 = “strongly agree.” Total scores ranged from 10 to 50, with higher scores indicating stronger TI. In this study, the scale showed good internal consistency, with Cronbach’s *α* = 0.921. The KMO value was 0.964. Confirmatory factor analysis supported good construct validity: *χ*^2^/df = 1.485, CFI = 0.988, TLI = 0.987, RMSEA = 0.020, and SRMR = 0.013. In addition, TI demonstrated satisfactory composite reliability and convergent validity (CR = 0.903, AVE = 0.564), with a square root of AVE of 0.751.

#### 3.2.4 Destination image

Destination image (DI) was measured using the scale developed by San Martín and Rodríguez del Bosque (2008), which was used to assess participants’ overall image perception of hiking destinations presented in SMHC. DI was conceptualized as a multidimensional construct consisting of cognitive and affective evaluations. The scale included five dimensions covering infrastructure, atmosphere, natural environment, affective image, and cultural environment. It comprised 16 items rated on a 7-point Likert scale ranging from 1 = “strongly disagree” to 7 = “strongly agree.” Total scores ranged from 16 to 112, with higher scores indicating a more positive DI. In this study, the scale demonstrated good internal consistency, with Cronbach’s *α* = 0.957. The KMO value was 0.986. Confirmatory factor analysis indicated good construct validity: *χ*^2^/df = 1.940, CFI = 0.975, TLI = 0.964, RMSEA = 0.042, and SRMR = 0.037. In addition, DI demonstrated satisfactory composite reliability and convergent validity (CR = 0.958, AVE = 0.585), with a square root of AVE of 0.765.

#### 3.2.5 Social connectedness

Social connectedness (SC) was measured using the scale developed by Lee and Robbins (1995). The scale assessed participants’ psychological sense of connection with others, groups, and the broader social environment, including feelings of interpersonal connection, social belonging, and alienation. Because all original items were negatively worded, all items were reverse-coded before statistical analysis. The scale included eight items rated on a 6-point Likert scale ranging from 1 = “strongly disagree” to 6 = “strongly agree.” After reverse coding, total scores ranged from 8 to 48, with higher scores indicating higher SC. In this study, the scale showed good internal consistency, with Cronbach’s α = 0.907. The KMO value was 0.945. Confirmatory factor analysis showed good construct validity: *χ*^2^/df = 2.181, CFI = 0.968, TLI = 0.967, RMSEA = 0.028, and SRMR = 0.022. In addition, SC demonstrated satisfactory composite reliability and convergent validity (CR = 0.907, AVE = 0.551), with a square root of AVE of 0.743. Detailed scale items and standardized factor loadings are provided in Supplementary Table S2.

### 3.3 Control variables

Gender, age, current status, occupational field, and monthly disposable expenses were included as control variables to account for demographic covariation in model estimation. These factors may be associated with Generation Z consumers’ social media use, leisure preferences, tourism consumption capacity, opportunities for hiking participation, acceptance of SMHC, and evaluation of DI. Accordingly, these variables were entered into the PROCESS regression models so that the focal direct, indirect, and interaction estimates were adjusted using the same covariate set.

### 3.4 Data processing and analysis

SPSS 26.0 and R were used for data processing and statistical analysis. Before the formal analyses, invalid questionnaires were removed, and reverse coding, total-score calculation, and variable standardization were completed. Total scores were calculated for SMHC, PI, TI, DI, and SC by summing the corresponding item scores. Except for demographic variables, all core variables entered into the PROCESS models were standardized using Z-scores. This procedure improved the comparability of path coefficients across scales with different response ranges and reduced potential multicollinearity in the interaction terms (Tabachnick and Fidell, 2019). The moderation terms were computed by multiplying the standardized predictor by the standardized moderator.

To provide additional evidence of measurement quality across the five constructs, composite reliability (CR) and average variance extracted (AVE) were also calculated for SMHC, PI, TI, DI, and SC. Harman’s single-factor test was used to assess common method bias (Podsakoff et al., 2003), followed by descriptive statistics and Pearson correlation analysis. Gender, age, current status, occupational field, and monthly disposable expenses were included as control variables. Hayes’ PROCESS Model 6 was used to test the serial mediating effects of TI and DI. Model 90 was used to examine the moderating role of SC and the moderated serial mediation effect (Hayes, 2022). All indirect effects were tested using 5,000 bootstrap resamples. Effects were considered significant when the 95% confidence interval did not include zero (Preacher and Hayes, 2008).

PROCESS analysis is suitable for testing mediation, moderation, and conditional process models, as well as for estimating bootstrap confidence intervals for indirect effects. Thus, it was aligned with the statistical aims of this study, which focused on serial mediation and moderated mediation. However, the PROCESS models were estimated using standardized observed composite scores rather than latent variables. Measurement error was therefore not explicitly modeled in the mediation and moderation analyses. Given the good internal consistency and construct validity of all scales, composite scores were used in the conditional process analyses.

Finally, network structure analysis was included as an exploratory analysis. R was used to construct an undirected node-level network including SMHC, TI, DI, and PI. This analysis aimed to identify conditional associations among items and dimensions (Epskamp et al., 2018). The significance level was set at *α* = 0.05.

## 4 Results

### 4.1 Common method Bias test

Because the data were mainly collected through self-report questionnaires at a single time point, Harman’s single-factor test was used to assess the potential influence of common method bias on the interpretation of the results. An unrotated exploratory factor analysis of all measurement items identified five factors with eigenvalues greater than 1, accounting for 61.725% of the total variance. The first factor explained 36.778% of the variance. These results indicate that no single factor dominated the total variance. However, Harman’s single-factor test is only a preliminary diagnostic and cannot establish the absence of common-method bias.

### 4.2 Descriptive statistics and correlation analysis

Table 2 presents the descriptive statistics and Pearson correlation results for the main variables. Overall, the directions of the correlations were consistent with theoretical expectations. Perceived SMHC was significantly and positively correlated with TI, DI, and hiking PI, with correlation coefficients of 0.508, 0.531, and 0.508, respectively, all reaching statistical significance (*p* < 0.001).

**Table 2:** Correlation and descriptive statistics.

Variables	*M*	*SD*	SMHC	TI	DI	PI	SC
SMHC	30.27	9.96	1				
TI	33.82	10.68	0.508***	1			
DI	74.51	24.08	0.531***	0.666***	1		
PI	16.37	5.41	0.508***	0.567***	0.631***	1	
SC	30.52	10.54	0.100***	0.121***	0.185***	0.186***	1

***p < 0.001.

TI was significantly and positively correlated with DI (*r* = 0.666, *p* < 0.001) and hiking PI (*r* = 0.567, *p* < 0.001). DI was also significantly and positively correlated with hiking PI (*r* = 0.631, *p* < 0.001). In addition, SC was significantly and positively correlated with SMHC, TI, DI, and hiking PI, with correlation coefficients of 0.100, 0.121, 0.185, and 0.186, respectively, all reaching statistical significance (*p* < 0.001).

### 4.3 Serial mediation effect test

#### 4.3.1 Regression results of the serial mediation model

Table 3 presents the regression results of the serial mediation model. SMHC was significantly and positively associated with TI (*β* = 0.51, *p* < 0.001). In the model with DI as the dependent variable, both SMHC (*β* = 0.26, *p* < 0.001) and TI (*β* = 0.54, *p* < 0.001) were significantly and positively associated with DI. In the model with PI as the dependent variable, SMHC (*β* = 0.19, *p* < 0.001), TI (*β* = 0.21, *p* < 0.001), and DI (*β* = 0.39, *p* < 0.001) were all significantly and positively associated with PI.

**Table 3:** Regression results for the serial mediation model.

Variable	*β* (TI) *SE*		*β* (DI) *SE*		*β* (PI) *SE*	
SMHC	0.513***	0.025	0.259***	0.024	0.194***	0.026
TI			0.536***	0.024	0.212***	0.030
DI					0.388***	0.030
Gender	−0.027	0.054	0.051*	0.045	−0.005	0.047
Age	−0.081**	0.033	−0.005	0.027	0.018	0.028
Current status	−0.010	0.018	−0.015	0.015	0.016	0.015
Field of study	−0.024	0.016	0.005	0.013	−0.011	0.014
Monthly disposable expenses	−0.055*	0.025	0.007	0.021	−0.004	0.022
*R^2^*	0.269		0.496		0.463	
*F*	72.242***		165.615***		127.030***	

*p < 0.05, **p < 0.01, ***p < 0.001.

In terms of effect size, the association between DI and PI was the strongest, followed by the associations of TI and SMHC with PI. This pattern suggests that, among Generation Z consumers, DI may be a more proximal evaluative variable related to outdoor hiking PI, whereas TI may represent an important psychological variable associated with subsequent destination evaluation.

#### 4.3.2 Results of the serial mediation effect test

As shown in Table 4, the total association between SMHC and PI was significant (*β* = 0.510, 95% CI [0.471, 0.548]), indicating that SMHC was positively associated with PI and supporting H1. After TI and DI were included in the model, the direct association between SMHC and PI remained significant (*β* = 0.194, 95% CI [0.143, 0.246]), suggesting that the relationship between SMHC and PI was partly consistent with indirect association pathways involving TI and DI. The total indirect association was 0.316, with a 95% CI of [0.278, 0.355], accounting for 61.9% of the total association. This pattern suggests that the relationship between SMHC and PI was not limited to their direct association, but was also consistent with indirect associations involving internal psychological evaluation variables.

**Table 4:** Total, direct, and indirect effects of SMHC on PI.

Effect	*β*	Boot *SE*	Boot 95% CI	Relative effect (%)
Total effect	0.510	0.019	[0.471, 0.548]	100
Direct effect	0.194	0.026	[0.143, 0.246]	38.1
Total indirect effect	0.316	0.020	[0.278, 0.355]	61.9
SMHC → TI → PI	0.109	0.016	[0.078, 0.140]	21.3
SMHC → DI → PI	0.100	0.012	[0.078, 0.126]	19.7
SMHC → TI → DI → PI	0.107	0.011	[0.086, 0.130]	20.9

For the specific pathways, the indirect association of SMHC → TI → PI was significant (*β* = 0.109, 95% CI [0.078, 0.140]), accounting for 21.3% of the total association. This suggests that TI was an important psychological variable involved in the association between SMHC and PI, supporting H2. The indirect association of SMHC → DI → PI was also significant (*β* = 0.100, 95% CI [0.078, 0.126]), accounting for 19.7% of the total association, indicating that DI was also involved in the indirect association pathway between SMHC and PI, supporting H3. Furthermore, the serial indirect association of SMHC → TI → DI → PI was significant (*β* = 0.107, 95% CI [0.086, 0.130]), accounting for 20.9% of the total association. Within the specified serial model, higher SMHC was associated with higher TI, higher TI with more positive DI, and more positive DI with stronger PI, supporting H4.

Overall, the three indirect pathways showed comparable effect sizes, suggesting that the association between SMHC and PI was not concentrated in a single indirect pathway. Instead, it was consistent with multiple indirect association patterns involving TI, DI, and their sequential linkage. The serial indirect estimate was statistically consistent with the theoretically specified ordering of TI, DI, and PI, although it does not establish temporal succession among these variables.

### 4.4 Moderating effect of SC and moderated serial mediation test

**Figure 2:**
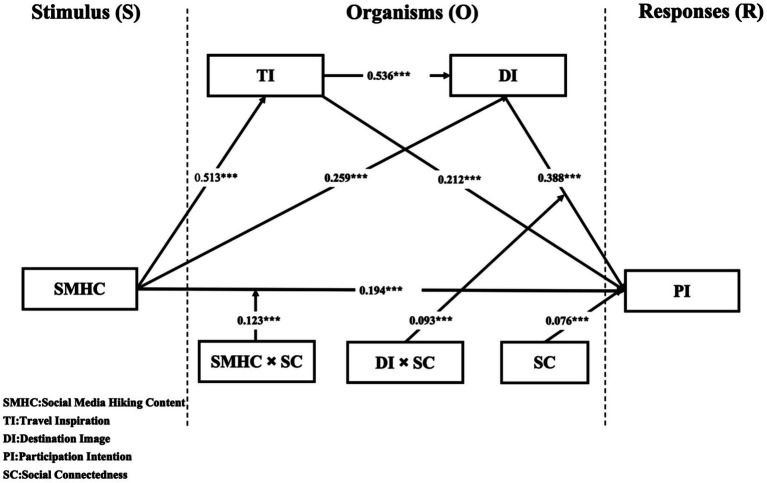
Final moderated serial mediation model with standardized path coefficients.

After identifying significant serial indirect associations, Hayes’ PROCESS Model 90 was used to examine whether SC moderated the associations between SMHC and PI and between DI and PI. The final model and standardized path coefficients are shown in Figure 2, and the regression results are presented in Table 5.

**Table 5:** Regression results for the moderating role of social connectedness.

Variable	*β*	*SE*	*t*	95% CI
SMHC	0.198***	0.026	7.756	[0.148, 0.248]
TI	0.155***	0.030	5.260	[0.097, 0.213]
DI	0.383***	0.029	13.056	[0.325, 0.440]
SC	0.076***	0.021	3.606	[0.035, 0.117]
SMHC × SC	0.123***	0.026	4.791	[0.073, 0.174]
DI × SC	0.093***	0.025	3.747	[0.044, 0.142]
Gender	−0.004	0.045	−0.209	[−0.098, 0.079]
Age	0.018	0.027	0.791	[−0.032, 0.075]
Current status	0.006	0.015	0.451	[−0.022, 0.035]
Field of study	−0.011	0.013	−0.841	[−0.037, 0.015]
Monthly disposable expenses	−0.004	0.021	−0.262	[−0.046, 0.035]
*R^2^*	0.502			
*F*	107.470***			

***p < 0.001.

The results showed that, in the regression model with PI as the dependent variable, SMHC (*β* = 0.198, *p* < 0.001), TI (*β* = 0.155, *p* < 0.001), DI (*β* = 0.383, *p* < 0.001), and SC (*β* = 0.076, *p* < 0.001) were all significantly and positively associated with PI. More importantly, the interaction term between SMHC and SC was significant (*β* = 0.123, *p* < 0.001), as was the interaction term between DI and SC (*β* = 0.093, *p* < 0.001). These results indicate that SC moderated both the SMHC–PI and DI–PI associations. Specifically, higher levels of SC were associated with stronger positive associations of SMHC and DI with PI. Thus, H5 and H6 were supported.

The moderation analysis further showed that the association between SMHC and PI varied across levels of SC. At low, medium, and high levels of SC, the conditional associations were 0.075, 0.198, and 0.321, respectively, and all were statistically significant. This suggests that, among individuals with higher SC, SMHC was more strongly associated with clear hiking PI.

For the moderated indirect associations, the SMHC → TI → PI pathway was significant (*β* = 0.080, 95% CI [0.049, 0.109]), but it did not vary across levels of SC. This indicates that SC did not significantly moderate the indirect association involving TI alone. By contrast, the SMHC → DI → PI pathway was more pronounced at higher levels of SC. The conditional indirect associations at low, medium, and high levels of SC were 0.075, 0.099, and 0.123, respectively, and the index of moderated mediation was significant (Index = 0.024, 95% CI [0.012, 0.039]). This result suggests that SC moderated the indirect association between SMHC and PI through DI.

The serial indirect association also showed a clear conditional pattern. The SMHC → TI → DI → PI pathway showed conditional indirect associations of 0.080, 0.105, and 0.131 at low, medium, and high levels of SC, respectively, and the index of moderated mediation was significant (Index = 0.026, 95% CI [0.012, 0.040]). These findings suggest that SC was associated with a stronger DI–PI association and a more pronounced serial indirect association involving SMHC, TI, DI, and PI. The conditional direct and indirect associations at different levels of social connectedness are presented in Table 6.

**Table 6:** Conditional effects by social connectedness.

Path/Effect	SC Level	*β*	Boot *SE*	Boot 95% CI	ModMed index [95% CI]
Direct effect (SMHC → PI)	−1 *SD*	0.075*	0.034	[0.009, 0.141]	
Direct effect (SMHC → PI)	Mean	0.198***	0.026	[0.148, 0.248]	
Direct effect (SMHC → PI)	+1 *SD*	0.321***	0.039	[0.245, 0.397]	
Ind1: SMHC → TI → PI		0.080***	0.016	[0.049, 0.109]	
Ind2: SMHC → DI → PI	−1 *SD*	0.075***	0.012	[0.053, 0.101]	0.024*** [0.012, 0.039]
Ind2: SMHC → DI → PI	Mean	0.099***	0.012	[0.076, 0.125]	
Ind2: SMHC → DI → PI	+1 *SD*	0.123***	0.016	[0.093, 0.156]	
Ind3: SMHC → TI → DI → PI	−1 *SD*	0.080***	0.012	[0.057, 0.103]	0.026*** [0.012, 0.040]
Ind3: SMHC → TI → DI → PI	Mean	0.105***	0.011	[0.085, 0.128]	
Ind3: SMHC → TI → DI → PI	+1 *SD*	0.131***	0.014	[0.104, 0.160]	

*p < 0.05, ***p < 0.001.

**Figure 3:**
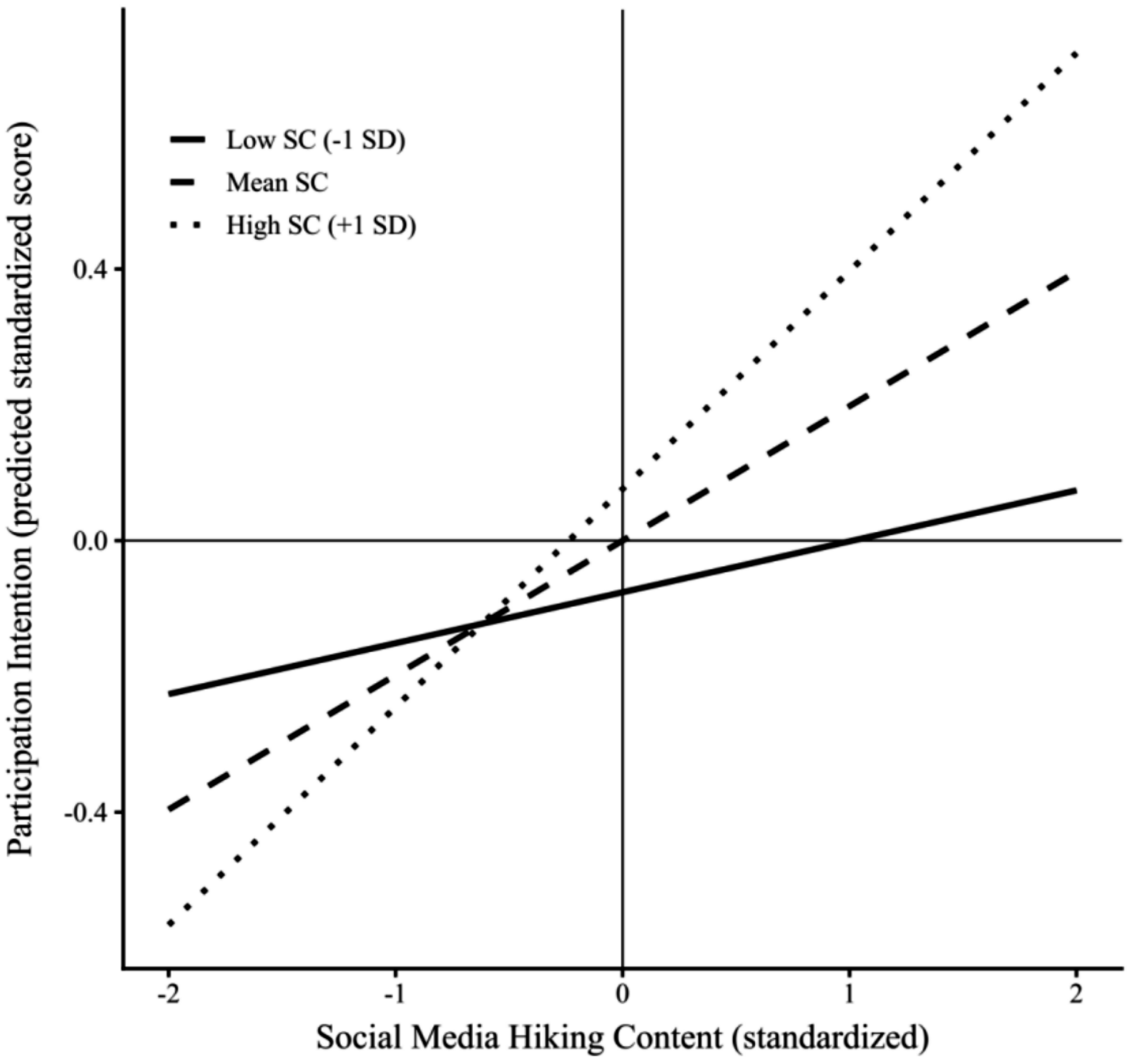
Moderating effect of SC on the relationship between SMHC and PI.

**Figure 4:**
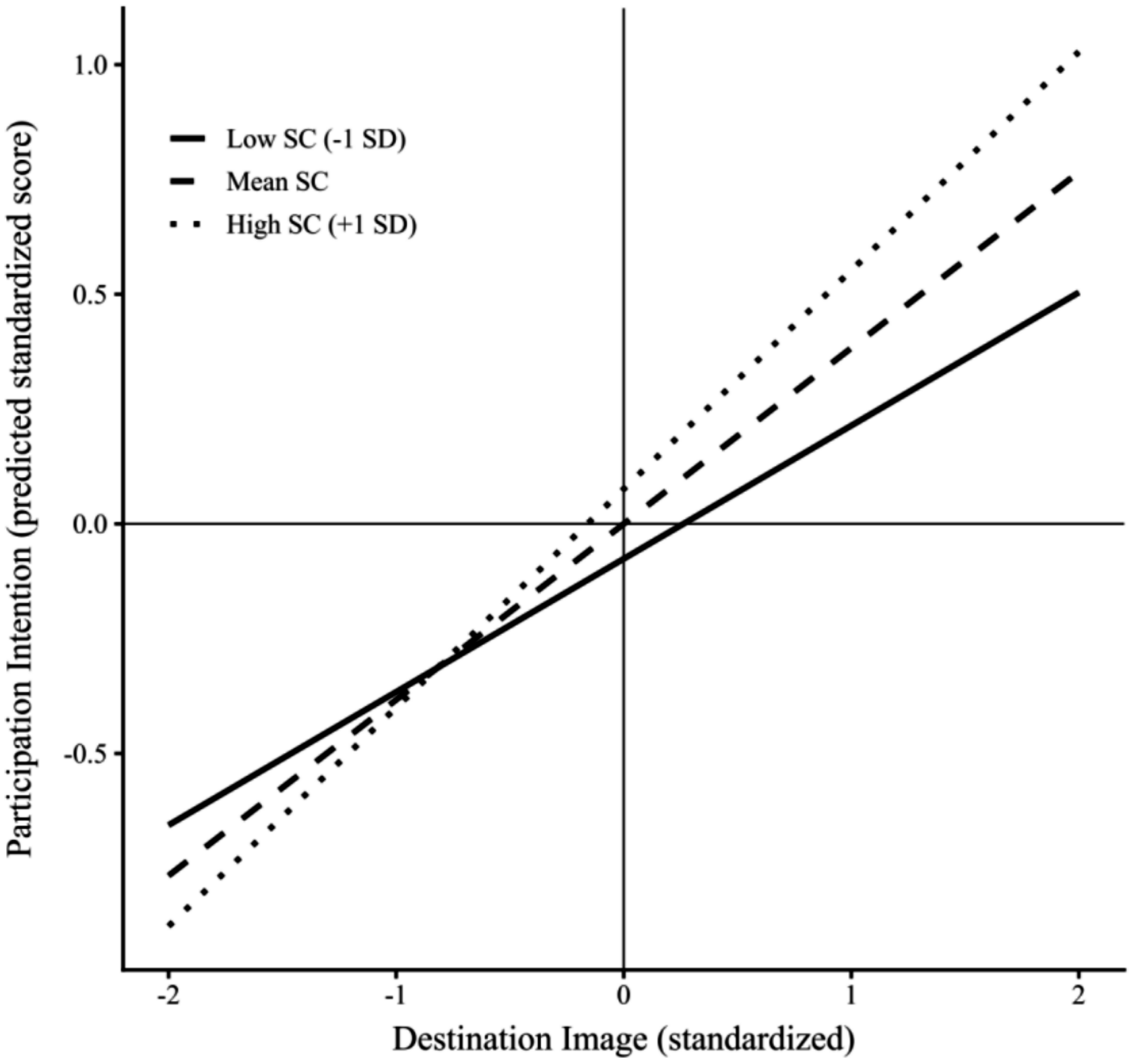
Moderating effect of SC on the relationship between DI and PI.

Figures 3, 4 further illustrate the conditional patterns identified in the simple slope analyses. For both the SMHC–PI and DI–PI associations, the slope was steeper at higher levels of SC than at medium or lower levels of SC. The three slopes showed a clear gradient, indicating that SC did not alter the direction of these associations but was linked to differences in their strength. Overall, higher SC was associated with stronger SMHC–PI and DI–PI associations and with more pronounced indirect association patterns involving DI and the TI–DI linkage. These findings suggest that, among Generation Z consumers, the associations among SMHC, psychological evaluation variables, and PI may be more evident when SC is higher.

### 4.5 Node-level network analysis

#### 4.5.1 Node coding and model specification

This study estimated an undirected network of 29 nodes to address two item-level questions that could not be answered by the composite-score PROCESS models: whether the associations among SMHC, TI, DI, and PI were distributed across their indicators or concentrated in particular components, and which items or dimensions formed the most prominent conditional connections across construct communities. The network analysis therefore moved beyond construct-level path estimation by identifying which specific items and dimensions carried the most prominent cross-community conditional connections among perceived content appraisal, psychological evaluation, and participation intention.

**Figure 5:**
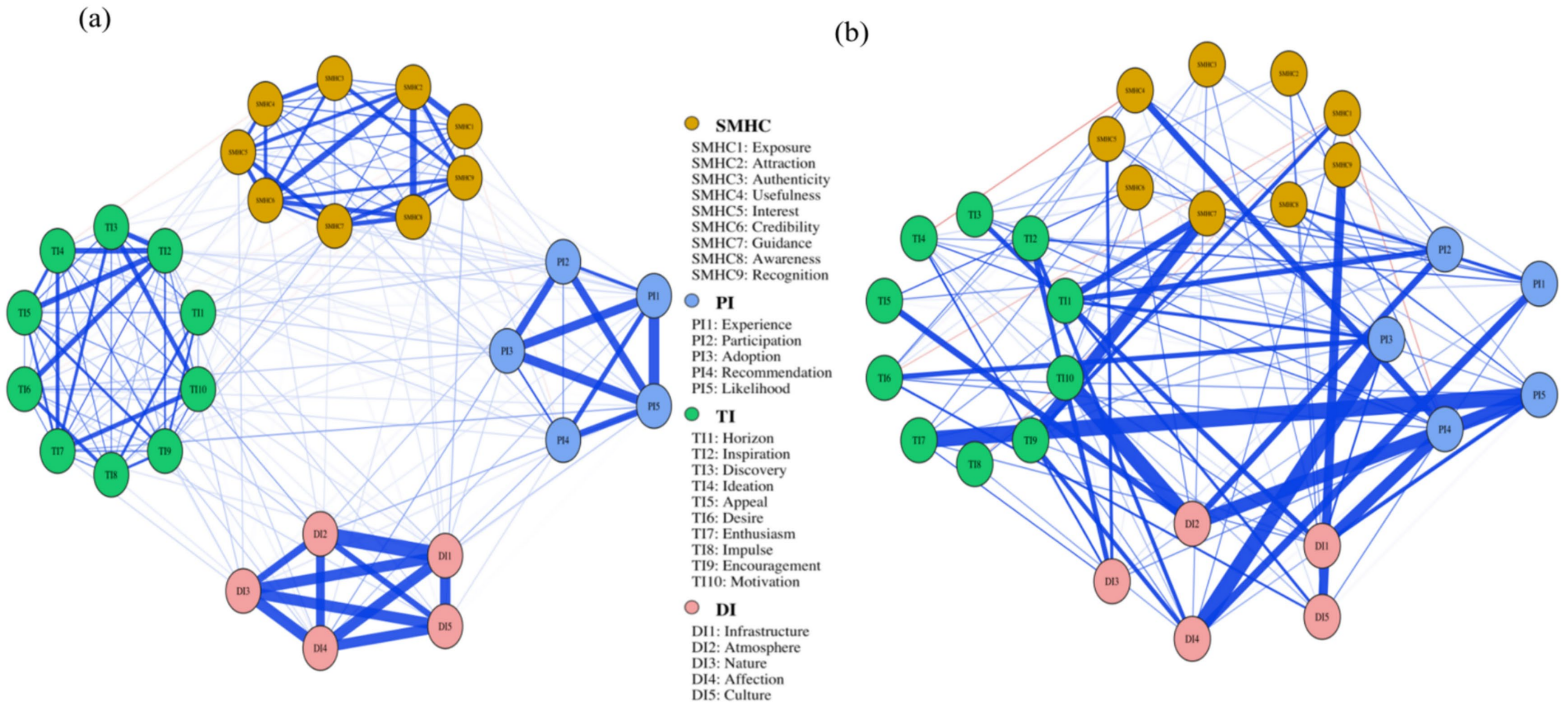
Node-level network structure. Panel **(a)** presents the full network, and panel **(b)** presents the bridge-only network. Blue and red edges indicate positive and negative associations, respectively.

The node-level network analysis focused on four focal variables. Specifically, SMHC included nine item nodes, TI included 10 item nodes, and PI included five item nodes. The original DI scale contained 16 items; however, because DI has a clear multidimensional structure, these items were integrated into five dimensional nodes according to their theoretical domains: DI1 = Infrastructure, DI2 = Atmosphere, DI3 = Nature, DI4 = Affection, and DI5 =  Culture. This approach preserved the theoretical structure of DI while preventing a single variable from occupying a disproportionate number of nodes in the network, thereby improving the interpretability of cross-community connections. The abbreviations and meanings of all nodes are shown in Figures 5a,b.

#### 4.5.2 Main network structure

As shown in Figures 5a,b, SMHC, TI, DI, and PI formed relatively clear internal clustering structures. The SMHC nodes were closely connected, suggesting that participants’ perceived evaluation of SMHC was reflected in a set of interrelated content features, including exposure, attraction, authenticity, usefulness, credibility, and guidance. The TI nodes also showed stable internal connections, indicating that TI was represented by multiple related components, such as broadened perspectives, idea generation, desire, impulse, and action motivation, rather than by a single item-level indicator.

The PI nodes were similarly concentrated, especially PI3, PI4, and PI5, which maintained relatively strong connections with other nodes. This pattern suggests that hiking PI was not limited to a general desire to participate but also included adoption intention, recommendation intention, and future participation likelihood. The DI nodes formed a relatively independent evaluation module, reflecting a cognitive–affective structure within the network. In terms of cross-community associations, the connections between PI and DI were the most prominent. PI5, PI3, and PI4 occupied central positions within the PI module and showed relatively strong associations with DI nodes. This pattern suggests that participation, adoption, and recommendation intentions were closely related to consumers’ overall evaluation of hiking destinations. Consistent with the preceding serial indirect association results, the network structure further indicates that DI may represent an important evaluative region linking psychological evaluation variables with PI.

#### 4.5.3 Node and bridge centrality analysis

As shown in Figures 5a,b, cross-community connections were mainly concentrated around PI and DI, indicating that these two domains served as key regions linking different psychological modules. Specifically, PI5, PI3, and PI4 were prominent in the bridge network, suggesting that future participation likelihood, adoption intention, and recommendation intention represented major PI-related bridge nodes. DI4, DI1, and DI2 also showed relatively strong bridge roles, indicating that affective image, infrastructure evaluation, and destination atmosphere were important evaluative cues associated with cross-community connections.

The bridge-only edge-weight matrix further showed that the strongest cross-community edge was PI3–DI4 (edge weight = 0.0588), indicating a close conditional association between adoption intention and affective image. This suggests that consumers’ tendency to include hiking in future leisure or travel choices was closely related to their affective evaluation of the destination. The second strongest cross-community edge was TI10–DI2 (edge weight = 0.0567), suggesting a conditional association between action motivation and perceived destination atmosphere.

**Figure 6:**
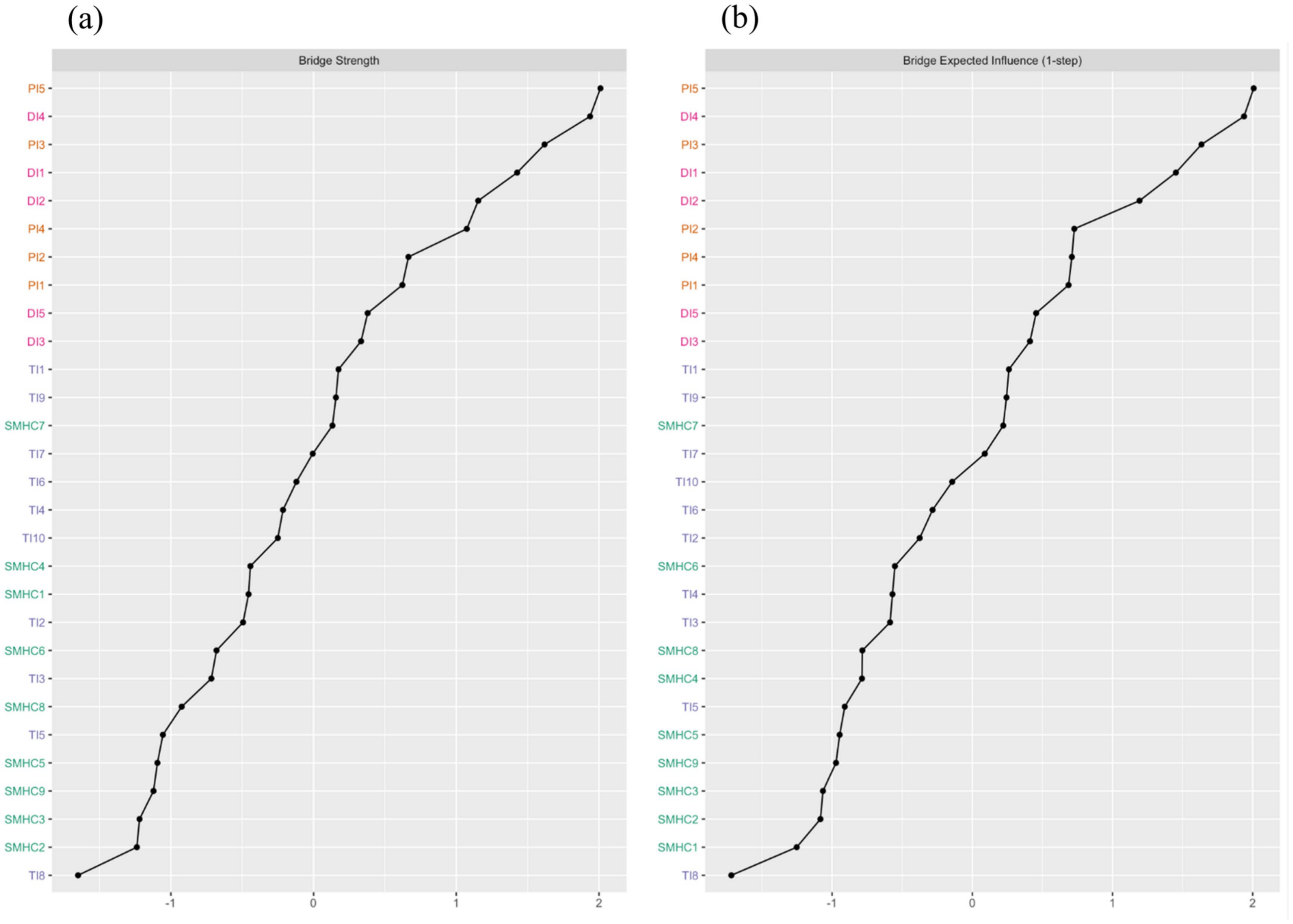
Panel **(a)** presents the bridge strength, and panel **(b)** presents the bridge expected influence.

As shown in Figures 6a,b, bridge centrality analysis indicated that PI5 had the highest bridge strength, followed by DI4, PI3, PI4, and DI1. This suggests that future participation likelihood, affective image, adoption intention, recommendation intention, and infrastructure evaluation played stronger cross-community connecting roles across variable groups. Bridge expected influence further showed that PI5 and DI4 had relatively high bridge influence, indicating that future participation likelihood and affective image were not only important within their own modules but also central in cross-dimensional associations. Overall, the node and bridge centrality results suggest that the association between SMHC and PI was not evenly reflected across all items and dimensions, but was more clearly concentrated around several bridge nodes, including future participation likelihood, affective image, adoption intention, recommendation intention, and infrastructure evaluation. These findings indicate that the cross-community association structure was not evenly distributed across all items and dimensions. Instead, it was concentrated around future participation likelihood, affective destination image, adoption intention, recommendation intention, and infrastructure evaluation. This localization cannot be obtained from composite-score PROCESS models and provides additional information about which components organize the broader association structure. The full bridge edge-weight matrix is presented in Supplementary Table S1.

#### 4.5.4 Network stability and interpretation

**Figure 7:**
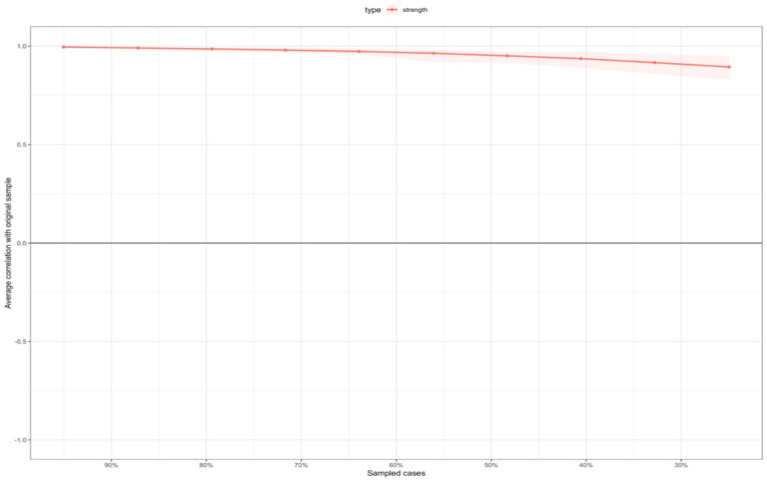
Case-dropping bootstrap analysis of strength centrality.

To further examine the robustness of the network results, supplementary centrality and bootstrap analyses were conducted, with the results presented in Supplementary Figures S1–S3. The results in Figure 7 showed that the estimated edge weights were generally distributed around the original sample estimates, and the strength index showed a certain degree of differentiation across nodes. This suggests that the network structure had acceptable stability. As these figures mainly served to support the robustness of the network estimation rather than directly test the theoretical hypotheses, they are presented as Supplementary materials.

## 5 Discussion

### 5.1 SMHC and PI among generation Z consumers

Within the present sample of Chinese Generation Z consumers with prior exposure to hiking-related social media content, SMHC was positively associated with PI (*β* = 0.510), supporting H1. After TI and DI were included, the direct SMHC–PI association was smaller (*β* = 0.194) and comparable to that reported between passive access to travel-related user-generated content and destination-selection intention (*β* = 0.166; Nguyen and Tong, 2023). This pattern indicates that the overall SMHC–PI association was substantial, whereas its unique direct component was more modest and much of the association was statistically consistent with the indirect pathways examined below.

The substantive relevance of this finding lies not in simply restating that social media is related to consumer intention, but in specifying this relationship in the context of hiking-related content and outdoor leisure participation (Kapoor et al., 2018; van Dijck and Poell, 2013). For potential Generation Z hiking consumers, participation often requires prior evaluation of scenery, routes, feasibility, physical demands, and contextual conditions. In this process, perceived SMHC may be understood as a content-based stimulus cue containing information and experiential references for evaluating hiking as a possible leisure activity. Previous studies have examined the association between social media use, tourism information quality, user-generated content, and travel or participation intention (Hung and Liou, 2023; Wang and Yan, 2022; Nguyen and Tong, 2023). However, less attention has been paid to hiking-specific social media content and its association with offline outdoor participation intention. Thus, the present study extends previous work by situating this relationship in the specific context of hiking.

Compared with general tourism information or user-generated content, SMHC combines route guidance, natural scenery, imagined bodily participation, and peer experience references. Its role may therefore be closer to a practical guide than to a simple information cue. SMHC contains not only destination information but also cues concerning how to arrive, how to participate, whether the activity is suitable, and whether the experience is worth sharing. This content profile is consistent with the comparatively strong SMHC–PI association observed in this study.

Node-level network analysis further enriches these analytical findings. In the bridge centrality results, PI5, PI3, and PI4 ranked among the important cross-community nodes, indicating that the association structure tended to converge on participation-related judgments, such as future participation, adoption, and recommendation. This is particularly relevant for Generation Z consumers because hiking is not merely a low-cost online preference, but a practical activity requiring time, physical effort, route understanding, and possible peer coordination. Therefore, when consumers perceive a clearer pathway for “how I can participate,” SMHC may be more closely associated with PI. Social practice theory similarly suggests that practices are organized through the integration of meaning, competence, and material conditions rather than by attitudes alone (Shove et al., 2012). The prominence of PI5, PI3, and PI4 suggests that PI represents an important bridge region between perceived content evaluations and possible practice adoption within the observed association structure.

Meanwhile, the strongest bridge edge, PI3–DI4, indicated that incorporating hiking into future leisure choices was closely related to affective attraction toward the destination (Hosany and Gilbert, 2010; Prayag et al., 2017). This finding suggests that Generation Z consumers’ willingness to adopt hiking as a leisure choice is not only associated with route information or landscape exposure, but also with whether the destination carries sufficient affective appeal (Van Boven and Gilovich, 2003; Belk, 2016). This is consistent with experiential consumption research, which emphasizes that consumers pursue not only functional benefits but also emotional, symbolic, and experiential value (Holbrook and Hirschman, 1982).

Overall, the network results help clarify the substantive meaning of H1. The association between SMHC and PI does not appear to depend on perceived content cues alone, but also on whether the content provides an understandable participation pathway and sufficient affective attraction (Pine II and Gilmore, 1998; Van Boven and Gilovich, 2003). For Generation Z consumers, the relevance of SMHC may lie not simply in seeing more hiking content, but in how such content presents hiking as a lifestyle practice that can be participated in, adopted, and shared (Munar and Jacobsen, 2014; Lo and McKercher, 2015). Accordingly, the association between SMHC and PI should be interpreted as an action-oriented association pattern that is consistent with the proposed SOR-based process, rather than as evidence of a causal sequence from perceived content evaluation to participation intention.

### 5.2 The serial mediation of TI and DI

After identifying the positive association between SMHC and PI, the serial mediation analysis indicated indirect association patterns involving TI and DI, supporting H2, H3, and H4. These findings suggest that the relationship between perceived SMHC and PI is not limited to participants’ evaluation of content attractiveness, authenticity, usefulness, credibility, or action guidance, but is also consistent with a differentiated psychological evaluation configuration. Perceived SMHC may contain cues about “how to participate,” yet these cues appear to be more closely linked to PI when they are accompanied by motivational and evaluative responses, namely TI and DI (Tussyadiah et al., 2018; Kim et al., 2020).

In this process, TI represents a proximal psychological response. Route narratives, natural scenes, peer experiences, and embodied participation images in SMHC may be associated with experiential imagination and action-oriented motivation among Generation Z consumers. Previous research has shown that tourism short videos are linked to travel intention through customer inspiration, indicating that inspiration-related appraisals may occupy a theoretically proximal position between digital content evaluation and travel intention (Wu and Ding, 2023).

The total indirect association (*β* = 0.316) accounted for 61.9% of the overall SMHC–PI association, with the three indirect pathways contributing in similar proportions. DI showed a stronger association with PI (*β* = 0.388) than TI (*β* = 0.212). Compared with previous research, the TI–PI association was weaker than that reported by Wu and Ding (2023), whereas the DI–PI association was broadly consistent with meta-analytic evidence of a moderate relationship (Afshardoost and Eshaghi, 2020). This pattern may reflect the practical demands of hiking: inspiration may coexist with uncertainty about destination infrastructure, atmosphere, and overall suitability. Practically, hiking-related content may be more relevant when it combines motivational appeal with information that supports concrete destination evaluation.

Thus, the serial mediation results do not simply indicate that TI and DI are useful variables; rather, they clarify their functional distinction within the proposed SOR-based process. Traditional SOR studies often treat the organism component as a single attitude, perception, or affective state, whereas the present findings are consistent with greater internal differentiation of the organism component: TI was associated with action imagination, whereas DI was associated with broader cognitive–affective destination appraisal. Research on virtual tourism similarly suggests that digital tourism stimuli are linked to visit intention through cognitive and affective responses, supporting the layered nature of the organism component (Kim et al., 2020). Accordingly, the contribution of this study to the SOR framework lies not in restating that stimulus-related cues are associated with responses, but in differentiating the organism component into inspiration-related motivation and destination appraisal.

Network analysis further specified this association pattern at the node level. The bridge edge TI10–DI2 showed a relatively strong cross-community connection, suggesting that action motivation is closely related to destination atmosphere (Borsboom and Cramer, 2013; Epskamp et al., 2018). Atmosphere may connect action motivation with embodied imagination because hiking is not a purely cognitive choice; it involves bodily movement, environmental immersion, fatigue expectation, and risk perception. A clearer destination atmosphere may help consumers mentally simulate the sensory and emotional experience of entering the setting, including air, light, natural sounds, relaxation, excitement, and safety (Rokka et al., 2023; Bitner, 1992). At the same time, DI nodes were further connected with PI, suggesting that DI is not merely a transitional variable in the serial mediation model, but also an evaluative hub closely linked to PI.

Specifically, SMHC is related not only to a desire to participate but also to action-oriented judgments that are conditionally associated with destination atmosphere, affective image, and practical accessibility (Bringmann et al., 2019; Jones et al., 2021). Therefore, the network results are consistent with the serial mediation findings by showing associations among SMHC, TI-related action imagination, DI-related evaluations, and PI. Together, the serial mediation and network results indicate a structured association pattern in which TI and DI represent differentiated and theoretically ordered psychological evaluation components.

### 5.3 Moderating role of SC

This study found that SC moderated both the SMHC–PI (*β* = 0.123) and DI–PI (*β* = 0.093) associations, supporting H5 and H6. These modest coefficients were smaller than those reported by Hung and Liou (2023). One possible explanation is that SC in the present study reflects a general sense of social belonging rather than hiking-specific companionship or support; it may be related to the social meaning assigned to content but does not directly capture the practical coordination required for participation. SC should therefore be interpreted as a boundary condition rather than a strong determinant of PI. In the Chinese context, this relational interpretation may be particularly relevant because social media practices among young consumers can combine information seeking with peer feedback, content sharing, and self-presentation. These practices may also help users maintain or extend social relationships around shared interests. Comparative evidence suggests that Chinese and American young users differ in their self-presentation strategies on social networking sites (Chu and Choi, 2010), indicating that the social meaning attached to SMHC may be culturally situated. This cultural and relational framing helps explain why SC may condition the two focal associations in different but theoretically coherent ways.

First, SC moderated the relationship between SMHC and PI. For individuals with higher SC, route information, peer experiences, and interactive feedback in perceived SMHC may be processed not only as informational cues but also as relational affordances. This interpretation is consistent with outdoor leisure research showing that the association between social media use and outdoor leisure PI was stronger at higher levels of social connection (Hung and Liou, 2023). It also echoes online travel community studies showing that social identification, community participation, and interaction with other members are associated with membership behavior and participation-related intentions (Qu and Lee, 2011; Casaló et al., 2010). Compared with these studies, the present findings suggest that SC is not only related to general social media use or online community participation but also conditions the association between perceived SMHC and PI. Hiking may therefore be understood not only as an object of personal interest but also as a lifestyle practice that can be shared, co-experienced, and incorporated into identity expression. In this sense, SMHC may be associated with relational imagination concerning peer participation, shared experience, and lifestyle belonging, making hiking participation a practice choice embedded in social relationships.

Second, SC moderated the relationship between DI and PI. DI reflects whether a place is perceived as attractive or worth visiting, whereas SC may further condition whether that place is imagined as suitable for shared experience and identity-related expression. Previous studies have shown that sharing travel experiences on social media is not merely an act of information dissemination but is also related to self-presentation, relationship maintenance, and social identification motives (Munar and Jacobsen, 2014; Lo and McKercher, 2015). For Chinese Generation Z consumers, hiking may therefore represent both a personal leisure choice and a socially visible practice through which shared experiences, relationships, and lifestyle identities are expressed. This interpretation is consistent with evidence that contemporary Chinese tourism values combine leisure and self-development with continuing family and relational orientations (Hsu and Huang, 2016). In this context, SC may be viewed as a factor linked to the ascription of social meaning. Accordingly, DI may be interpreted not only as an individual destination evaluation but also in relation to relational interaction and lifestyle identification. This interpretation is consistent with the finding that the positive association between DI and PI was stronger among participants with higher levels of SC.

Furthermore, within the indirect association patterns, moderation was concentrated in pathways involving DI rather than in the TI-only pathway. This pattern suggests that TI is closer to immediate psychological activation, whereas the association between DI and PI may depend more strongly on the social meaning assigned to destination evaluations. In other words, SC may be less closely related to whether consumers report feeling inspired by SMHC, while still conditioning the association between destination-related evaluations and participation judgments. Liu et al. (2024) similarly found that social media affordances were associated with cognitive and affective destination-image formation through social presence and parasocial interaction. This finding is consistent with the present interpretation that the DI–PI association may become more salient when destination evaluations are embedded in stronger relational contexts.

Taken together, the moderating role of SC may be culturally situated. Within the present Chinese sample, SMHC and DI may be interpreted not only as sources of personal information and evaluation but also as resources for shared experience, peer recognition, and lifestyle identification. This relational meaning is consistent with the stronger associations of SMHC and DI with PI at higher levels of SC.

## 6 Limitations and future directions

This study has several limitations. First, the cross-sectional design limits the ability to determine temporal ordering or causal relationships among the variables. Although the proposed model was developed within the SOR framework, the findings should be interpreted as conditional associations consistent with the theoretical model rather than as causal evidence. Future research could use longitudinal, experimental, ecological momentary assessment, or platform-based behavioral designs to examine the dynamic relationships among SMHC, psychological evaluations, and actual hiking participation. For example, experimental studies could expose participants to different types of hiking-related content to test how specific content features are associated with psychological evaluations and participation intention. Longitudinal studies could track SMHC contact, perceived content evaluations, and later hiking participation over time. Platform-based studies could further incorporate behavioral indicators such as browsing, likes, saves, comments, and sharing records to improve ecological validity.

Second, all focal variables were measured using self-report questionnaires administered in a single online survey, creating potential risks of recall bias and common-method bias. Several procedural measures were used to reduce these risks, including anonymous participation, standardized instructions, and an explicit statement that there were no right or wrong answers. Harman’s single-factor test did not identify a dominant single factor; however, this procedure cannot rule out common-method variance. Because the questionnaire did not include a theoretically unrelated marker variable and no common latent method factor was estimated, the observed associations may partly reflect the shared measurement context and should therefore be interpreted cautiously. Future studies should incorporate temporal or source separation, marker variables, common latent factor models, platform-based behavioral data, or objective records of hiking participation to provide more robust assessments of common-method bias.

Third, the serial mediation and moderation analyses were based on standardized observed composite scores. Although all scales showed acceptable internal consistency and structural validity, PROCESS estimates models using composite scores and does not explicitly model measurement error. Future studies could use latent variable structural equation modeling or latent moderated structural equation modeling to test the robustness of the relationships among the variables.

Fourth, this study used the SOR framework to organize the relationships among SMHC, psychological evaluations, and PI. However, the SOR framework mainly provides a broad “stimulus–organism–response” explanatory structure. It does not fully account for factors such as platform algorithms, content creator characteristics, offline accessibility, and actual outdoor participation conditions. Future research could integrate the SOR framework with other theoretical perspectives, such as social influence theory, the theory of planned behavior, or technology acceptance models, to provide a more comprehensive explanation of the relationship between SMHC and PI.

Fifth, the generalizability of the findings should be interpreted cautiously. Although this study focused on Generation Z consumers with prior exposure to hiking-related social media content, participants were recruited through convenience sampling, mainly from university networks, digital platforms, and hiking-related groups. Therefore, the findings should not be generalized to the entire Chinese Generation Z population, but are more applicable to the surveyed Generation Z consumers who had previously encountered SMHC. Future studies could use probability or stratified sampling and compare different countries, generational groups, exposed and non-exposed consumers, and outdoor activity contexts. Latent class analysis or multi-group analysis could also be used to examine whether the proposed model applies differently across consumer segments.

Sixth, cultural orientations were not directly measured, and no cross-national comparison was conducted. Therefore, the culturally contextualized interpretations of SC should be regarded as plausible, context-sensitive explanations rather than evidence that the observed moderating pattern is unique to Chinese culture. Chinese Generation Z consumers should also not be treated as a culturally homogeneous group. Future studies could directly measure individual-level cultural orientations and use cross-national or multi-group designs to examine whether the proposed relationships vary across cultural and platform contexts.

Finally, the network analysis used an undirected cross-sectional network. Therefore, the edges and bridge nodes should be interpreted as conditional associations among items and dimensions rather than as causal pathways. Future research could use longitudinal or dynamic network models to examine whether key nodes predict later changes in actual hiking participation.

## 7 Conclusion

Within the present convenience sample of Chinese Generation Z adults aged 18–28 who had previously encountered hiking-related social media content, perceived SMHC was positively associated with PI. The observed SMHC–PI relationship was statistically consistent with separate and serial indirect associations involving TI and DI. SC moderated the SMHC–PI and DI–PI associations, which were stronger at higher levels of SC. The node-level network further identified PI and DI as prominent cross-community regions, with PI5, DI4, PI3, PI4, and DI1 emerging as important bridge nodes.

Overall, these findings characterize the correlational associations among perceived SMHC, motivational responses, destination evaluation, SC, and hiking PI within the surveyed sample. They do not establish temporal or causal relationships and should not be generalized to the entire Chinese Generation Z population. Within these boundaries, the findings suggest that hiking-related social media communication may consider experience-based content, clear participation cues, affective destination appeal, infrastructure information, and consumers’ relational contexts.

## Data Availability

The raw data supporting the conclusions of this article will be made available by the authors, without undue reservation.

## Supplementary material

SUPPLEMENTARY FIGURE S1 Strength centrality of all nodes in the estimated network.

SUPPLEMENTARY FIGURE S2 Bootstrap analysis of edge-weight accuracy.

SUPPLEMENTARY FIGURE S3 Bootstrap difference test for strength centrality.

SUPPLEMENTARY TABLE S1 Bridge edge-weight matrix for cross-community associations among SMHC, TI, DI, and PI nodes.

SUPPLEMENTARY TABLE S2 Scale items and standardized factor loadings.
