# Supplementary figures and images for: From digital stimulus to hiking participation: an SOR-based examination of the internal associations between social media hiking content and participation intention in Chinese generation Z consumers
Source: Front Psychol. 2026 Jul 20;17:1877548. doi: 10.3389/fpsyg.2026.1877548 (PMC13429592; doi:10.3389/fpsyg.2026.1877548)

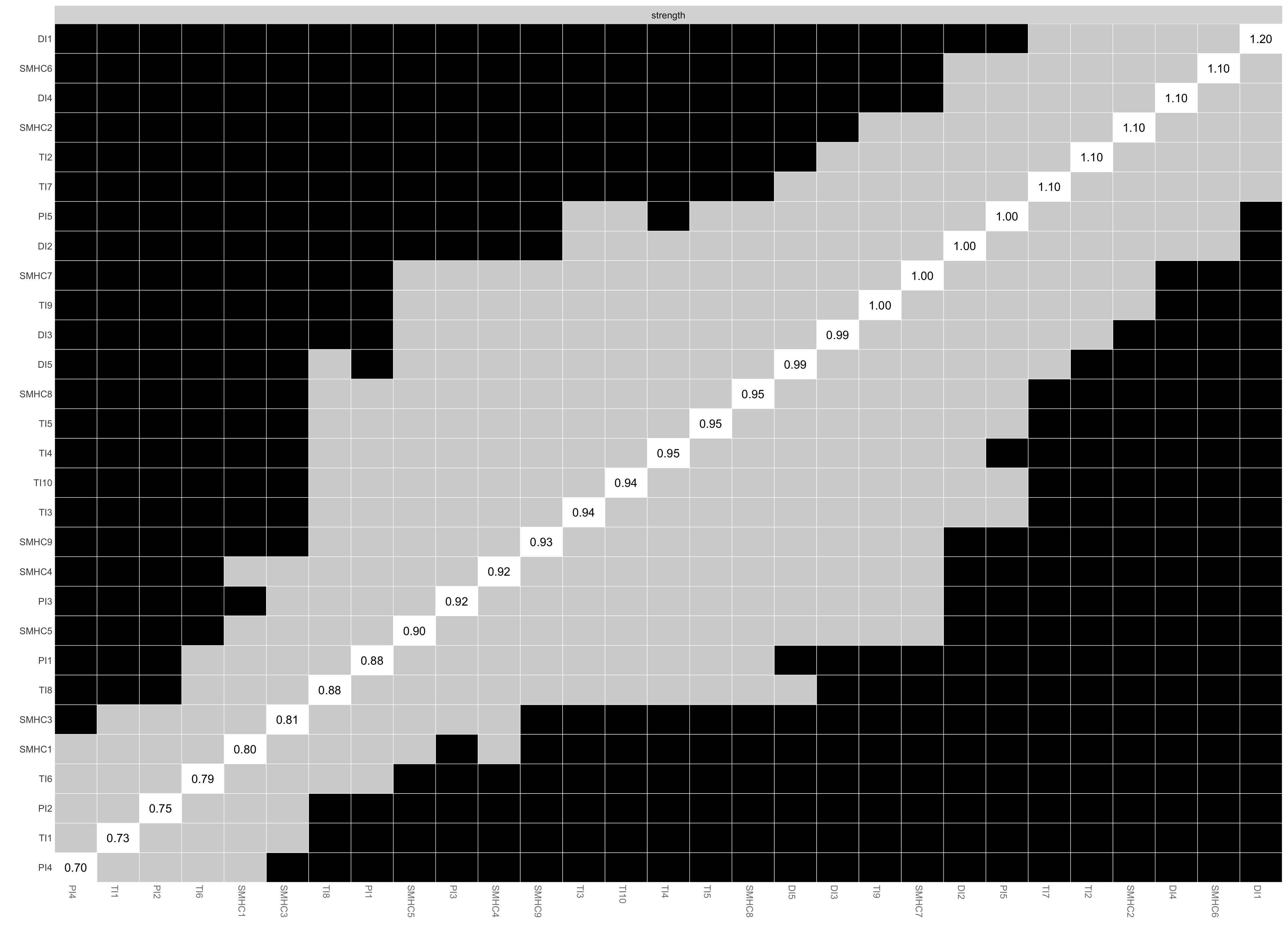

Supplement: SUPPLEMENTARY FIGURE S1 — Strength centrality of all nodes in the estimated network. [file Image_1.PNG]

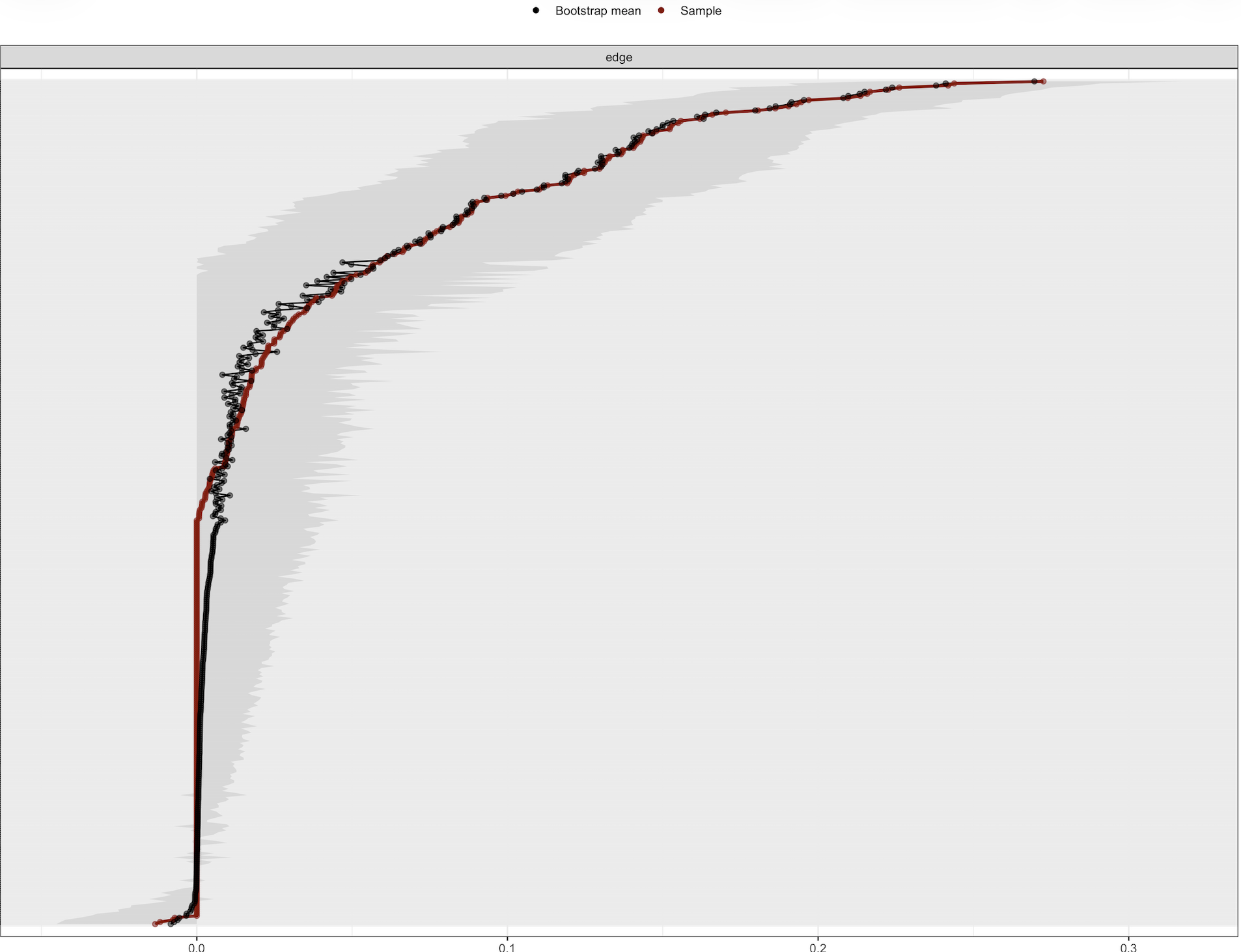

Supplement: SUPPLEMENTARY FIGURE S2 — Bootstrap analysis of edge-weight accuracy. [file Image_2.PNG]

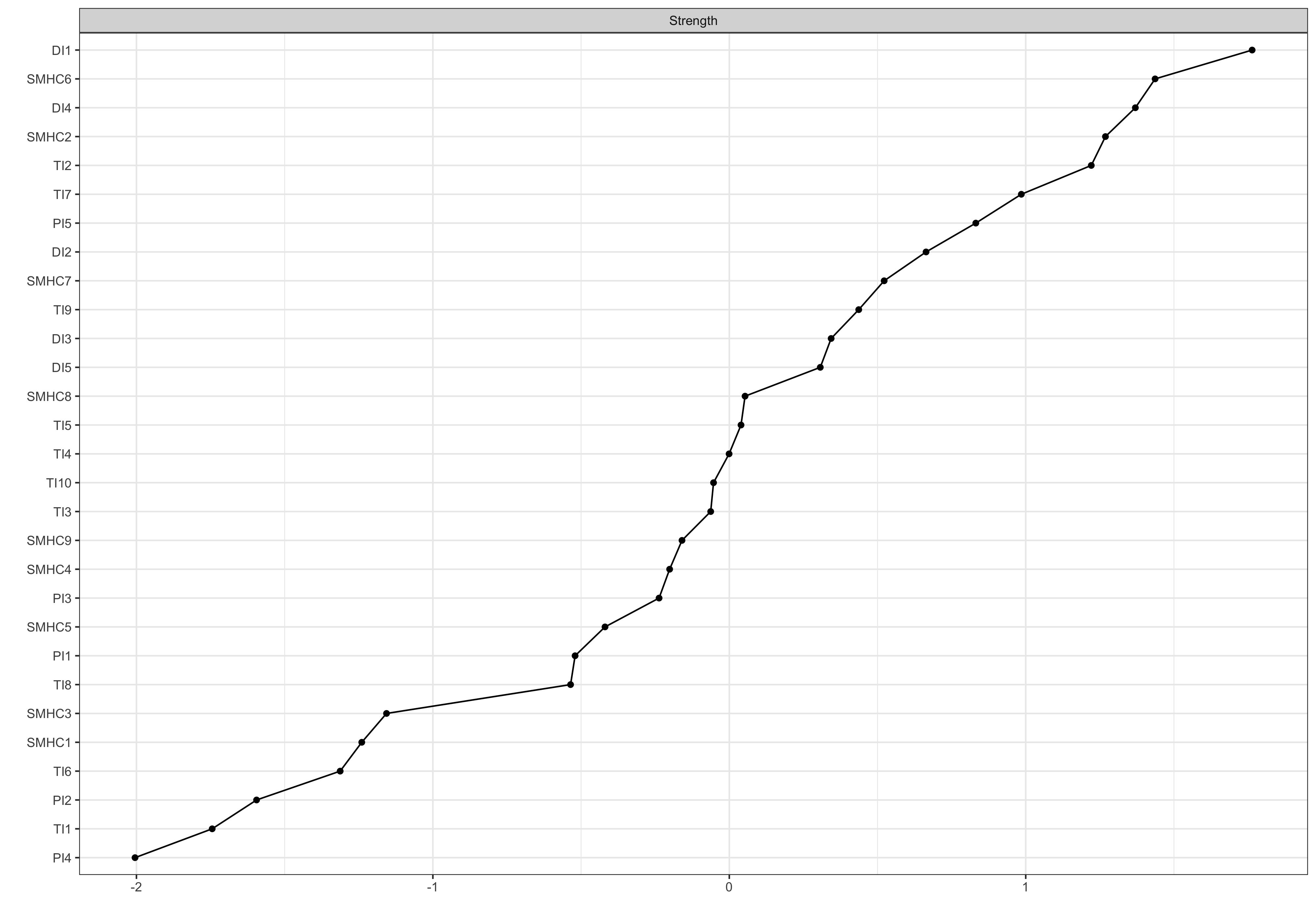

Supplement: SUPPLEMENTARY FIGURE S3 — Bootstrap difference test for strength centrality. [file Image_3.PNG]
